# Impact Analysis of Standardized GNSS Receiver Testing against Real-World Interferences Detected at Live Monitoring Sites

**DOI:** 10.3390/s19061276

**Published:** 2019-03-13

**Authors:** Mohammad Zahidul H. Bhuiyan, Nunzia Giorgia Ferrara, Amin Hashemi, Sarang Thombre, Michael Pattinson, Mark Dumville

**Affiliations:** 1 Finnish Geospatial Research Institute, National Land Survey of Finland, 02430 Kirkkonummi, Finland; giorgia.ferrara@nls.fi (N.G.F.); amin.hashemi@nls.fi (A.H.); sarang.thombre@nls.fi (S.T.); 2 Nottingham Scientific Limited, Nottingham NG7 2TU, UK; michael.pattinson@nsl.eu.com (M.P.); mark.dumville@nsl.eu.com (M.D.)

**Keywords:** Global Navigation Satellite Systems (GNSS), radio frequency interference, receiver test standard

## Abstract

GNSS-based applications are susceptible to different threats, including radio frequency interference. Ensuring that the new applications can be validated against the latest threats supports the wider adoption and success of GNSS in higher value markets. Therefore, the availability of standardized GNSS receiver testing procedures is central to developing the next generation of receiver technologies. The EU Horizon2020 research project STRIKE3 (Standardization of GNSS Threat reporting and Receiver testing through International Knowledge Exchange, Experimentation and Exploitation) proposed standardized test procedures to validate different categories of receivers against real-world interferences, detected at different monitoring sites. This paper describes the recorded interference signatures, their use in standardized test procedures, and analyzes the result for two categories of receivers, namely mass-market and professional grade. The result analysis in terms of well-defined receiver key performance indicators showed that performance of both receiver categories was degraded by the selected interference threats, although there was considerable difference in degree and nature of their impact.

## 1. Introduction

Global Navigation Satellite System (GNSS) technology plays an important role in an ever-expanding range of safety, security, business, and policy critical applications. Moreover, many critical infrastructures, such as telecommunication networks, energy grids, stock markets, etc. rely, at least to some degree, on uninterrupted access to GNSS positioning, navigation, and timing services [1]. At the same time however, different threats have emerged which have the potential to degrade, disrupt, or completely deny these GNSS services without prior warning [2]. This paper focusses on the threat posed by Radio Frequency Interference (RFI) to the GNSS L1/E1 spectrum. RFI can be emitted either unintentionally, e.g., by commercial high-power transmitters, ultra-wideband radar, television, VHF, mobile satellite services, and personal electronic devices, or intentionally, e.g., by jammers and more sophisticated signal spoofing devices [2,3]. 

Due to the proliferation of GNSS-based applications to almost every part of the globe it can be assumed that this phenomenon of RFI threat to GNSS is no longer seen as a local or regional problem, but rather requires an international perspective. To ensure GNSS is protected, there is now a need to also respond at an international level by ensuring that there is the following: (*i*) A common standard for real-world GNSS threat monitoring and reporting, and (*ii*) a global standard for assessing the performance of GNSS receivers and applications under threat. 

GNSS threat reporting standards would allow for compilation of real-world threats into a database that could be analyzed to develop GNSS receiver testing standards that ensure new applications are validated against the latest threats [4,5,6,7]. Both these standards are currently missing across all civil application domains and, if implemented, could potentially open the field for wider adoption and success of GNSS in the higher value markets.

The EU Horizon2020 research project STRIKE3 (Standardization of GNSS Threat reporting and Receiver testing through International Knowledge Exchange, Experimentation and Exploitation) is a European initiative that addresses the need to monitor, detect, and characterize GNSS threats and to propose standardized testing procedures to validate different categories of receivers against these threats [4,5,6,7]. Importantly, STRIKE3 has deployed an international network of GNSS interference monitoring sites that monitor interference on a global scale and capture real-world threats for further analysis [8]. 

Utilizing thousands of threats collected from this network over a three-year period, STRIKE3 has developed a baseline set of typical real-world interference/jamming threats that can be used to assess performance of different categories of GNSS receivers. This baseline set, which is used in this paper to assess the performance of two categories of receivers, consists of five different threat signatures as follows: Wide swept frequency with fast repeat rate, multiple narrow band signals, triangular and triangular wave swept frequency, and tick swept frequency. Additional details about the behavior of these five threat signatures in the time and frequency domain and the criteria for their selection can be found in [9]. The STRIKE3 project also proposed standardized testing procedures for GNSS receivers, which make use of these threat signatures for receiver validation. Collectively, the above activities aim to improve mitigation and resilience of future GNSS receivers against real-world interference threats. An example of such an attempt has already been made in [3], where an adaptive interference mitigation technique was proposed to counteract unintentional Narrow-Band Interference (NBI) detected at one of the STRIKE3 monitoring sites. 

In [10], European Telecommunications Standards Institute’s (ETSI) Satellite Communication and Navigation (SCN) group of the Technical Committee Satellite Earth Stations and Systems (TC SES) suggested to elaborate a firm and common standard for GNSS-based location systems. The group addresses interference from the perspective of robustness to interference, interference localization, and GNSS denial survival in addition to formulating location systems’ minimum performance requirements. In particular, [11] discusses the GNSS based applications and standardization needs more inclusively. In addition, the Resilient Navigation and Timing Foundation urges for development of standards for interference-resistant receivers to include ARAIM (Advanced Receiver Autonomous Integrity Monitoring) and RAIM to protect, toughen, and augment GNSS [12]. The international aviation community has also been considering updating standards regarding GNSS aviation receivers, improving their ability to withstand interference from repeaters, pseudolites, and jammers [13]. 

In order to address the need from various GNSS stakeholders, the STRIKE3 consortium proposed a draft standard for GNSS receiver testing against real-world interferences detected at different STRIKE3 monitoring sites, along with a test campaign following the guidelines from the drafted receiver test standard. This paper discusses how the receiver testing is done and presents some of the results obtained within STRIKE3 testing activity. In particular, the paper addresses mass market and professional grade receiver testing. The test platform and the test methodology are described in Section 2 and Section 3, respectively. Section 4 provides an overview of the receiver configuration and the simulated GNSS scenario settings, whereas Section 5 presents the five threats chosen for the tests. Results of the tests carried out are presented and analyzed in Section 6 and, finally, conclusions are drawn in Section 7.

## 2. Test Platform

This Section describes the physical platform used to test mass-market and professional grade GNSS receivers. For both categories, the test approach is the following:

1. The first iteration of the testing is performed using a clean GNSS signal without the presence of any interference threats. This helps establish the baseline performance of the Receiver Under Test (RUT).
2. Each subsequent test case addresses one threat scenario (one type of interference signal). Thus, the performance of the receiver is recorded sequentially against the different interference signals.
3. Analysis of the recorded results is carried out to show the receiver behavior under conditions of the different interference signals in terms of pre-defined performance metrics. This performance is compared to the baseline under nominal signal conditions.

**Figure 1:**
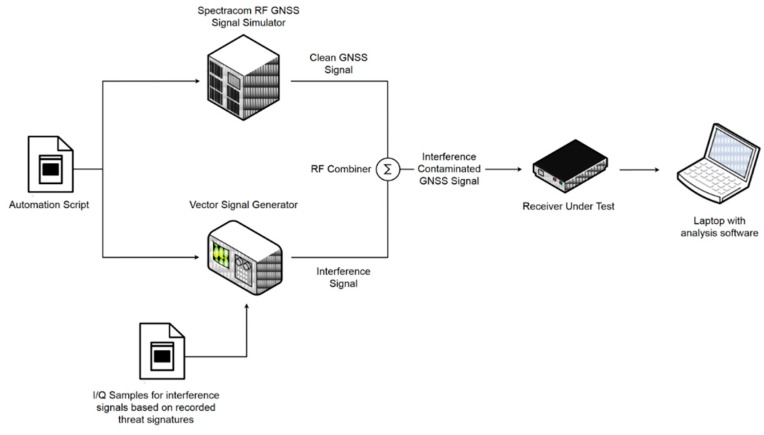
Receiver test platform.

The test set-up, which is shown in Figure 1, includes the following equipment:

1. Spectracom GSG-6 RF GNSS constellation simulator [14],
2. Keysight Vector Signal Generator N5172B [15],
3. RUT, and
4. Laptop for data storage and analysis.

In addition, generic components such as coaxial cables and signal combiners are used. The clean GNSS signal is generated from a multi-constellation, multi-frequency Spectracom GSG-6 hardware simulator, whereas the threat signature is generated using Keysight’s Vector Signal Generator (VSG) [15] through the replay of raw I/Q (In-phase/Quad-phase) sample data. The raw I/Q data are captured in the field from the STRIKE3 monitoring sites and are used as input to the VSG, which then re-creates the detected threat by continuously replaying the data in a loop. Both the GNSS signal simulator and the VSG are controlled via software in order to automate the testing process. The automation script is used to control these devices remotely and to limit human intervention. The script also provides synchronization between the two instruments in order to ensure repeatability of the tests and reliability of the results. A laptop is used to record and analyze the performance of the receiver against the different threat signals. The analysis is performed using a MATLAB-based script that processes the NMEA output messages from the RUT.

## 3. Receiver Test Methodology

Three different test methodologies are performed for each receiver category. A brief overview of each test methodology is presented as follows. 

### 3.1. Baseline Test Method

A clean GNSS signal, in the absence of interference, is fed to the RUT to validate its performance under nominal condition. The total duration of this test is 60 min.

### 3.2. Time to Recompute Position (TTRP) Solution Test Method

**Figure 2:**
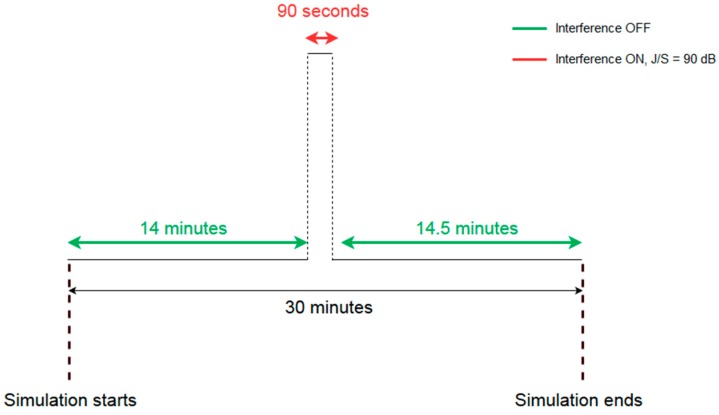
TTRP test profile.

This test is used to measure the time taken for the RUT to recover immediately after a strong interference event. The interference is switched on 14 min after the simulated scenario starts and it is applied for 90 s. The interference power is fixed to a value such that the receiver loses position solution. In the tests, the interference power corresponds to a Jamming-to-Signal (J/S) ratio of 90 dB. The time taken between switching off the interference source and the first fix is recorded as the TTRP, after interference. The profile of this test methodology, whose total duration is 30 min, is illustrated in Figure 2.

### 3.3. Sensitivity Test Method

**Figure 3:**
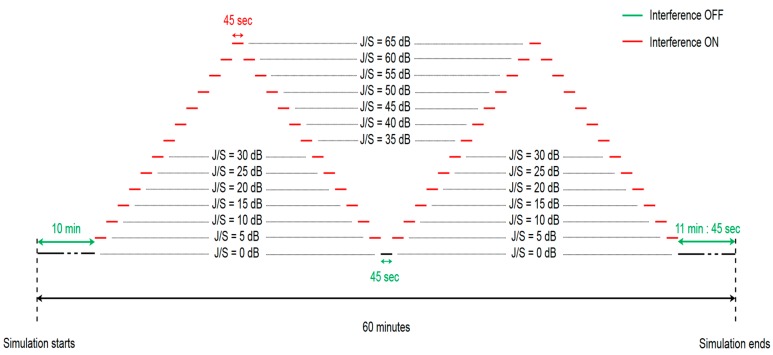
Sensitivity test profile.

This test is conducted by varying the power of the interfering signal. The interference is turned on 10 min after the simulation starts and it follows a two-peak ramp power profile. The initial interference power is such that J/S is 5 dB and then the interference power is increased by 5 dB every 45 s until reaching the first power peak, which corresponds to a J/S of 65 dB. After it reaches the first peak, the interference power is decreased. The power step and the dwell duration per power step are again, respectively, 5 dB and 45 s. The entire process is repeated a second time. The profile of this test methodology, whose total duration is 60 min, is illustrated in Figure 3.

In order to assess the performance of the RUT in the presence of interference, different metrics are observed. The following outputs from the GNSS receiver are recorded and analyzed for all the test methodologies:

1. Number of tracked satellites;
2. Position fix indicator;
3. Number of satellites used in fix;
4. Carrier-to-Noise density (C/N_0_) ratio;
5. East-North-Up deviations of the receiver’s solution from the true position.

Moreover, depending on the test methodology, additional parameters are evaluated. In the case of TTRP test method, the time taken for the RUT to re-obtain a position fix after a strong interference event is measured. Other metrics are of interest in the case of the sensitivity test method. In particular, the Jamming-to-Signal ratio at which position solution is no longer available and the availability of the position solution during the interference event are computed in the sensitivity tests. Furthermore, the maximum horizontal and vertical position errors are computed for the interval in which the interference is present when the receiver offers a valid position fix.

## 4. Receiver Configuration and Simulated Scenario Settings

For the sake of simplicity, the GNSS receiver configuration (as defined in terms of the constellation and frequency bands to be used in the validation) is restricted to GPS L1 single frequency (GPS SF) and Galileo E1 single frequency (GALILEO SF). The RUT was first configured in factory settings mode and then all the necessary modifications, based on the requirements of each individual test case scenario, were applied. It must be noted, however, that the test procedure and set-up is also equally applicable to other receiver configurations. 

Since the RUT compensates for the atmospheric effects, atmospheric modelling capability is required in the testing activity. The GNSS simulator supports a number of models to simulate such effects and, in the tests, Klobuchar and Saastamoinen models were used for the ionosphere and the troposphere, respectively. The receiver was configured in static stand-alone mode. Table 1 provides an overview of the simulated scenario settings, including also the start time, the duration, the GNSS signal power, and the interference power levels for the different test methodologies.

When performing the tests, an elevation mask and a C/N_0_ mask are applied for the receiver’s PVT computation. Satellites with elevations lower than 5° as well as satellites whose C/N_0_ are lower than 25 dB-Hz are excluded from position computation, as reported in Table 2. At the beginning of every test, the RUT is in cold start mode.

## 5. Real-World Interference Classification

The purpose of the proposed test standards is not to propose a fixed set of threats to cover all types of signal, but instead to develop draft standards for testing receivers against interference that has been observed in the real world through the dedicated interference monitoring network. Using real interference threats that have been detected in the field allows interested parties (e.g., certification bodies, application developers, receiver manufacturers, etc.) to better assess the risk to GNSS performance during operations and to develop appropriate counter-measures. However, with many thousands of potential threats being detected by the monitoring network, it is impractical to test receivers against all detected threats. Therefore, an initial selection process has been done and a baseline set of 5 types of threats has been selected for inclusion in the draft test standard for receiver testing [9]. 

In particular, each receiver is tested against the following 5 types of interference threats, described in Table 3: Wide swept frequency with fast repeat rate, multiple narrow band signals, triangular and triangular wave swept frequency, and tick swept frequency. Each of these threats is generated by the VSG replaying raw I/Q data captured in the field during a real event, if not mentioned otherwise. In Table 3, each type of threat is explained via an example plot with two figures. The left figure denotes the estimated power spectral density at the GNSS L1/E1 carrier frequency ±8 MHz and the right figure represents the spectrogram of the perceived interference. As can be seen in the example plots, the spectrogram color map is defined so that the ‘blue’ color represents the least signal power, whereas the ‘red’ color represents the most signal power. 

## 6. Test Results

This section presents the results of the standardized tests for the two categories of receivers, Mass-Market (MM) and PROfessional (PRO) RUT. An initial version of the test results was published in [16], where the results were analyzed only for one interference signature. The published article in [16] only offered a general overview on the impact on the receivers when exposed to real-world interference and it lacked a thorough analysis of receivers’ performance under different circumstances (e.g., impact of C/N_0_ thresholding, impact of how the interference signal is generated, impact of various interference signatures, impact on dual frequency receiver, etc.). Therefore, an extensive result analysis of receiver testing is presented as follows. 

For each RUT category, a summary table provides a comparison between the impacts of the 5 selected types of interference. In particular, the results are shown in terms of the following metrics:

1. Maximum horizontal position error during the interference interval;
2. Maximum vertical position error during the interference interval;
3. Position fix availability during the interference interval;
4. Jamming-to-Signal ratio at which the position fix is lost for at least 5 s (J/S_PVT_lost_);
5. Jamming-to-Signal ratio at which the position fix is reobtained for at least 5 s (J/S_PVT_reobtained_);
6. TTRP.

The receiver’s performance under conditions of the different interference signals is compared to the baseline under nominal signal conditions. For the baseline case, the statistics are computed by considering the interval corresponding to the one when, in the interference test case, the interference would be on. This ensures that the differences in the test statistics of the receiver performance, between a baseline test case and the selected threat test case, is solely due to the impact of the threat signature on the GNSS signal. 

### 6.1. Mass Market Receiver

**Figure 4:**
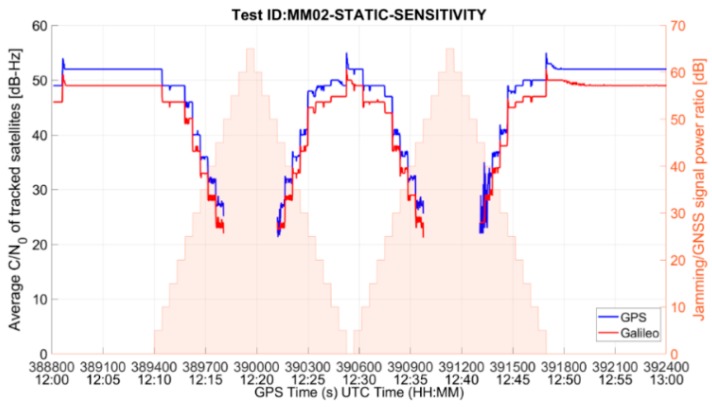
Average C/N_0_ of tracked satellites for MM RUT in the presence of threat signature 02.

**Figure 5:**
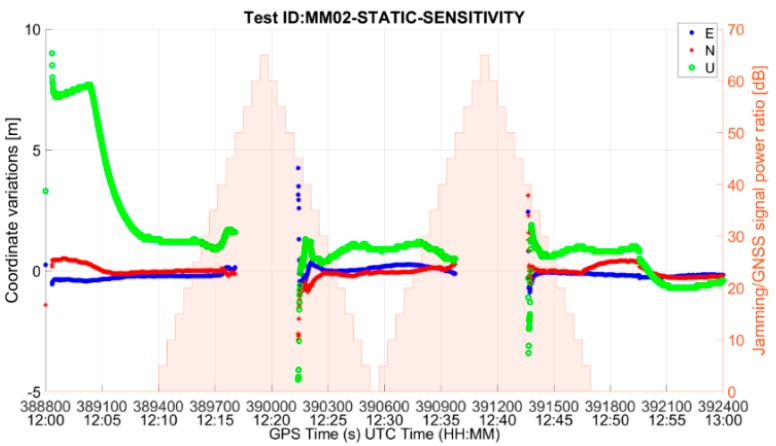
East-North-Up deviations for MM RUT in the presence of threat signature 02.

The performance of the MM RUT is summarized in Table 4. Whenever an interfering signal is present, as its power increases, the receiver performance degrades until, at some point, the position fix is lost. The receiver is then capable to re-compute a position solution only when the interference power decreases. An example of such interference impact on the mass-market RUT performance is given in Figure 4 and Figure 5, which show, respectively, the average C/N_0_ of tracked satellites and the East-North-Up deviations of the position solution for the test case MM02-STATIC-SENSITIVITY (threat signature 02: multiple narrowband interfering signals). In both figures, the corresponding two-peak ramp interference power profile could also be seen (in the right-hand Y-axis) for the sensitivity test methodology, as described in Section 3.3.

The inaccurate position solution, especially in the vertical component computed by the RUT at the beginning of the test, is due to the cold start and the resulting unavailability of ionospheric parameters and to the convergence of the navigation filter.

As it can be seen from Table 4, the Jamming-to-Signal ratio at which the position fix is lost for at least 5 s (J/S_PVT_lost_) and the Jamming-to-Signal ratio at which the position fix is reobtained for at least 5 s (J/S_PVT_reobtained_) are in the range 45–50 dB and 30–45 dB, respectively. This translates into a position fix availability in the presence of the five interference threats between ~60% and ~70%. The triangular swept frequency interference signature (i.e., test case MM03) seems to be the most impactful, both in terms of availability and maximum position error. 

The TTRP tests showed that the mass-market RUT is capable of recovering from a strong interference event almost immediately. The TTRP value is, in fact, 1 s for four of the tested interference signatures, and 4 s in the case of a wide swept frequency signal with a fast repeat rate (i.e., test case MM01).

### 6.2. Professional Grade Receiver

**Figure 6:**
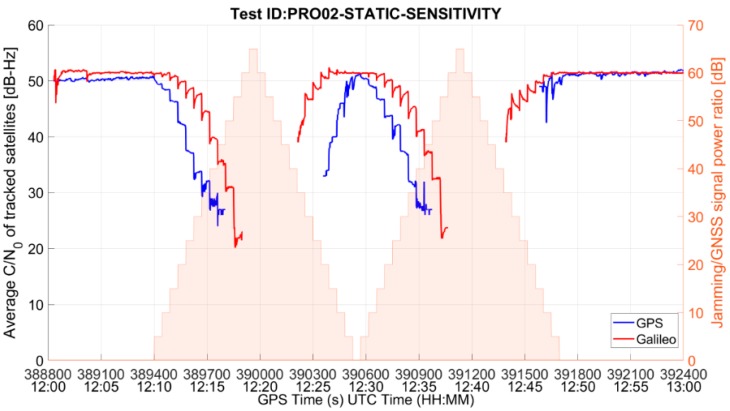
Average C/N_0_ of tracked satellites for PRO RUT in the presence of threat signature 02.

**Figure 7:**
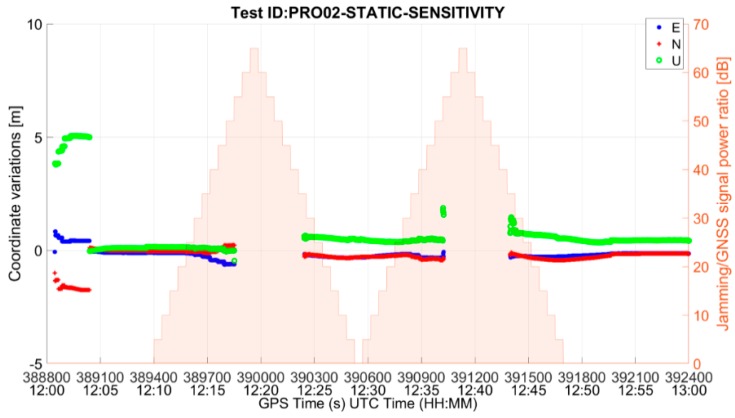
East-North-Up deviations for PRO RUT in the presence of threat signature 02.

Similar to the MM receiver, the consequences of the interference presence on the PRO grade RUT are degradation in the signal quality and hence the position accuracy, which worsen as the interference level increases, until the RUT loses its position fix. An example of such interference impact on the PRO grade RUT is given in Figure 6 and Figure 7, which show the average C/N_0_ of tracked satellites and the East-North-Up deviations of the position solution for the test case PRO02-STATIC-SENSITIVITY (threat signature 02: multiple narrowband interfering signals), respectively.

Additionally, in this case, due to the cold start and the resulting unavailability of ionospheric parameters and to the convergence of the navigation filter, the professional grade RUT offers an inaccurate position solution in the beginning, especially in the vertical component.

Table 5 summarizes the results for the PRO grade RUT. It can be seen that both J/S_PVT_lost_ and J/S_PVT_reobtained_ are in the range 40–55 dB and 35–45 dB, respectively. Consequently, the position fix availability for the professional grade RUT in the presence of the five interference threats is between ~61% and ~66%. The TTRP tests showed that the professional grade RUT takes from 6 to 10 s to recover from a strong interference event.

### 6.3. Comparison Between Mass Market and Professional Grade RUT with Default C/N_0_ Masking Settings

From the comparison between Table 4 and Table 5, it can be observed that the availability of the position fix during the interference interval does not differ much between mass market and professional grade receivers. This is partly due to the fact that the C/N_0_ mask was set to 25 dB-Hz for both the receivers’ PVT computation. In order to properly compare the behavior of the two categories of RUT, additional tests with default C/N_0_ mask settings were carried out. In particular, sensitivity tests were performed for the mass market and the professional receiver in the presence of the threat signature 03, triangular chirp swept frequency interference signal (MM03 and PRO03).

**Figure 8:**
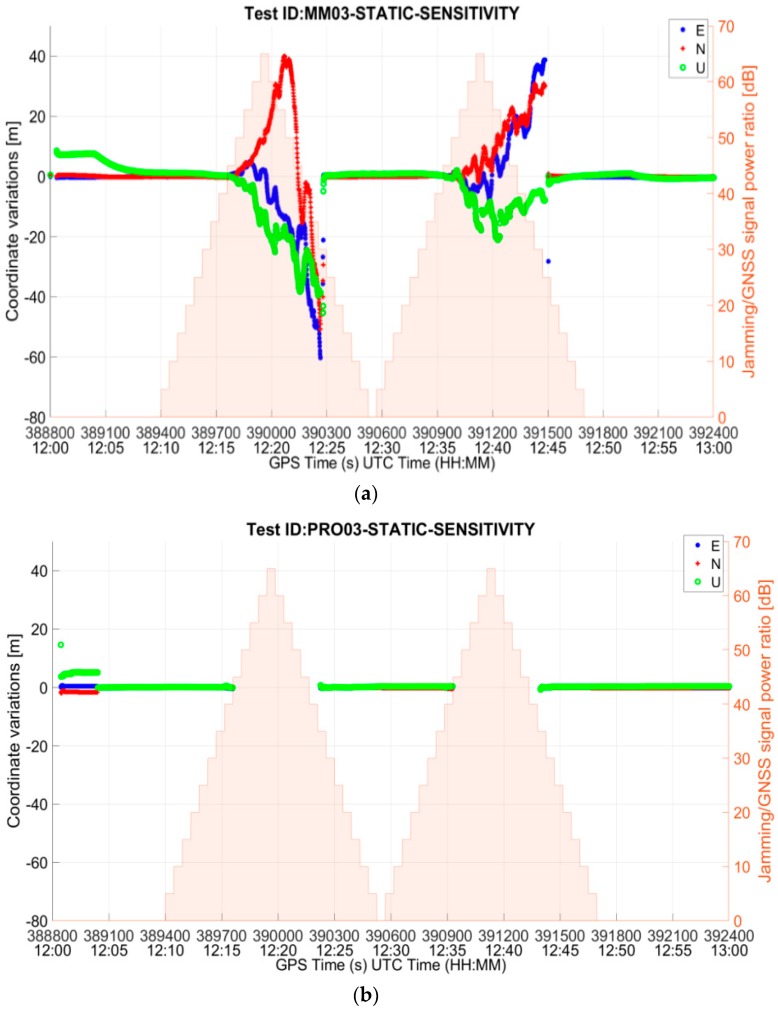
East-North-Up deviations for (**a**) MM RUT and (**b**) PRO RUT in the presence of threat signature 03 when C/N_0_ default settings are used in both the receivers.

Figure 8 shows the ENU (East-North-Up) deviations of the position solution for the MM and the PRO receivers. As it can be seen from the figure, the behaviour of the two receiver categories differs significantly when default settings of C/N_0_ are used. In particular, the MM RUT prioritizes the availability of the position solution over its accuracy. During the interference interval, there are only a few epochs at which the receiver does not yield a position solution, but this high yield comes with degraded positioning accuracy. On the other hand, the PRO RUT prioritizes the accuracy over the availability. It does not offer the position solution as often during the interference interval, but when it does the position errors are small.

**Figure 9:**
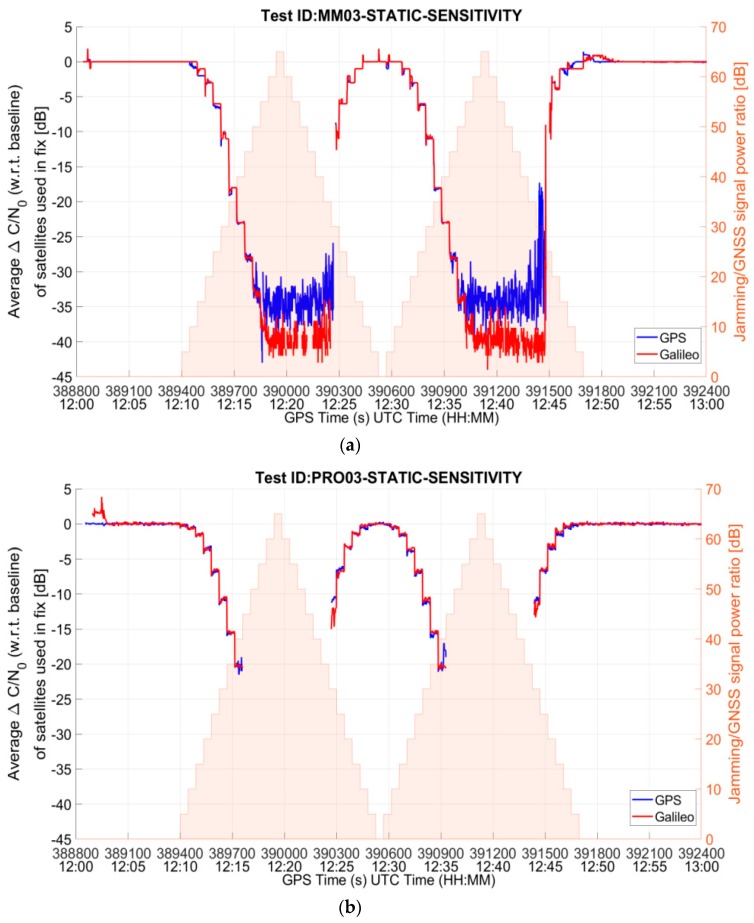
Drop in average C/N_0_ for (**a**) MM RUT and (**b**) PRO RUT in the presence of threat signature 03 when C/N_0_ default settings are used in the receivers.

The difference between the mass market and the professional grade receivers’ behavior is also visible in Figure 9, which shows the drop in the average C/N_0_ of the satellites used in position fix with respect to the baseline for the entire duration of the test. While the MM RUT continues to use very low quality signals in order to provide a position solution, even if inaccurate, for as long as possible, the PRO RUT stops computing the solution when the signal quality decreases by about 20 dB.

A summary of the results of the tests with default C/N_0_ settings is given in Table 6. The maximum horizontal and vertical errors are computed for the interval in which the interference is present and the receiver also offers a position fix. As already discussed, the position fix availability during the interference interval for the mass-market receiver is high (97.91%) at the expense of position accuracy. On the other hand, the professional grade RUT preserves the position accuracy at the expense of solution availability (58.58%). The maximum horizontal and vertical errors in the test case are only slightly larger than in the baseline case. It is important to recall here that the availability is computed only for the duration when interference is active. It can be observed from Table 6 that, when manufacturer’s default receiver settings are used, the mass market RUT has much higher sensitivity as compared to the professional grade RUT. The former is able to withstand the interference event through the entire rising ramp of the interference power profile and for most of the falling ramp duration. The position fix is lost after the interference power peak has been reached (precisely, at J/S = 30 dB) and it is regained after a few seconds. The behavior is different for the PRO RUT which instead stops offering a position solution as soon as the interference reaches a power level such that J/S is 45 dB.

### 6.4. Synthetic vs. Real-World Signature Impact Analysis

The results presented in the previous sections were obtained by generating the interference through the replay of raw I/Q sample data captured in the field by the STRIKE3 monitoring sites. In addition, a set of tests was also conducted using a second approach for the threat generation; creating synthetic I/Q data with properties that are representative of the real signals and replaying them with the VSG. The advantage is that the generated signal is free of multipath and GNSS PRN codes, hence it is cleaner than the one recorded in the field. However, this approach has limitations when the original signal is complex and difficult to re-create synthetically, such as in the case of chirp tick signal, as the resultant interference signal may not accurately reflect the real one and, therefore, the impact on the receiver performance may be different. 

Sensitivity testing with synthetic interference signals was performed only for the MM RUT. In particular, the following four, out of the five threat signatures described in Table 3, were possible to be re-created via VSG: Wide swept frequency with fast repeat rate, multiple narrow band signals, triangular, and triangular wave swept frequency. The results are summarized in Table 7, which also includes the results obtained with live interference data recorded in the field. For each performance metric, the column with label “S” contains the results for synthetic signature, whereas the column with label “R” contains the results for recorded signature. 

**Figure 10:**
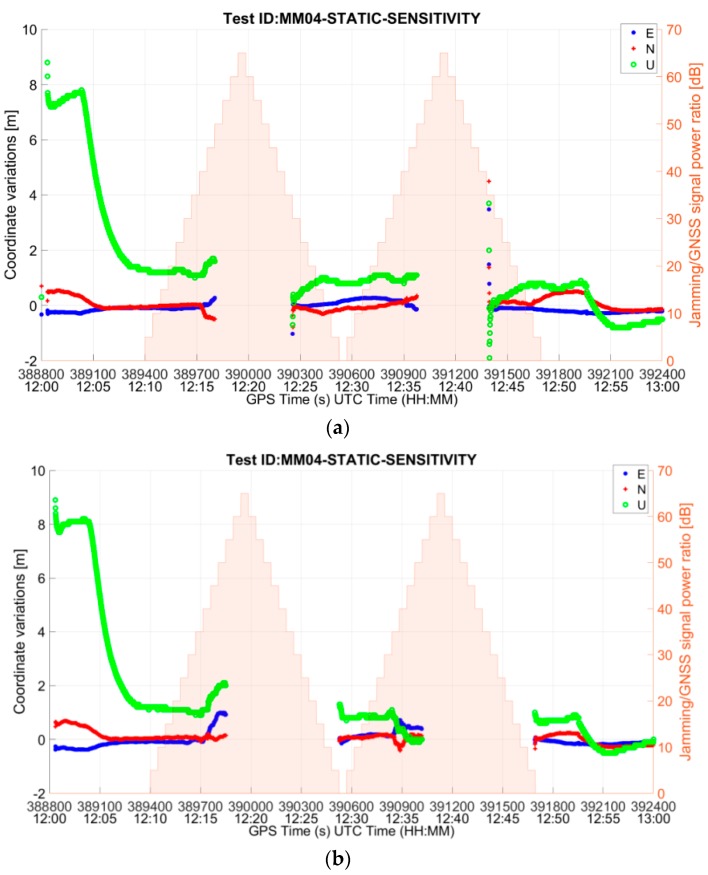
East-North-Up deviations for test case MM04-STATIC-SENSITIVITY using (**a**) recorded interference and (**b**) synthetic interference.

As it can be observed from Table 7, the synthetic signatures have a stronger impact on the RUT. This is shown by the reduced availability of the position fix due to the longer time needed by the receiver to recover from the interfering event. J/S_PVT_reobtained_ takes values in the range of 30–45 dB when recorded interference is used, while it is in the range of 0–25 dB in the case of synthetic threat signatures. In one test case (i.e., triangular wave swept frequency signal, MM04), the RUT is capable of reobtaining the PVT solution only after the interference has been turned off. As an example, the difference in the impact to the receiver between the recorded and synthetic signature can be seen in Figure 10, which shows the East-North-Up deviations of the receiver’s solution from the true position in the test case MM04, using recorded interference and synthetic interference.

It is observed that the number of epochs in which the RUT is not capable of providing a position fix is significantly higher when synthetic interference is used. This could be due to the fact that the synthetic signatures are free of multipath and GNSS PRN codes and hence are much cleaner than the recorded ones. This is indeed an empirical conclusion from the perceived tests, which would require further investigation in this direction. It would always remain a challenge to reproduce a real-world detected interference event, since the reproduction of the signal will always be based on the digitized raw In-phase Quad-phase (IQ) samples in the presence of a real GNSS signal. On the other hand, the reproduction of a synthetically generated signal does not have any GNSS signature in it, as the samples are taken from the real jamming device and then reproduced via VSG. 

### 6.5. Dual Frequency Receiver Testing

Dual-frequency tests, where the RUT is configured to receive GPS/Galileo signals, in both L1/E1 and L5/E5 frequency bands, were also performed. In particular, a dual-frequency professional grade receiver was assessed in the presence of the wide swept frequency with fast repeat rate interfering signal (i.e., PRO01). The interference was generated only at the L1/E1 carrier frequency. The objective of such test was to investigate if the RUT could intelligently exploit the frequency diversity in the presence of interference in one of the frequency bands. 

**Figure 11:**
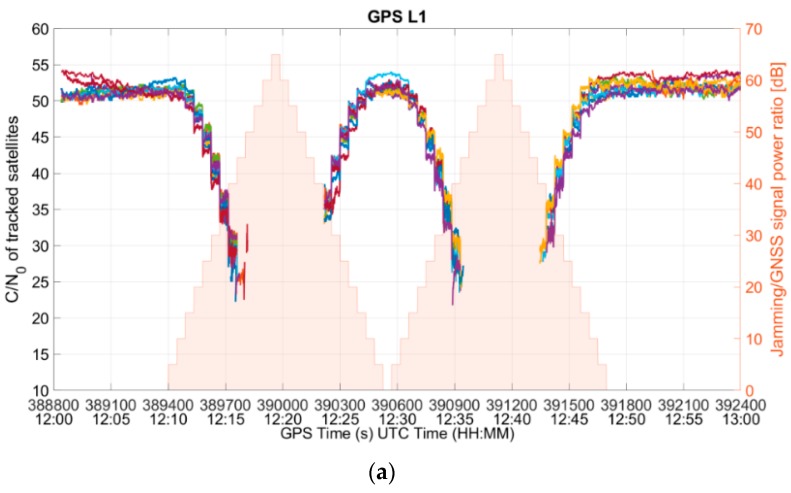

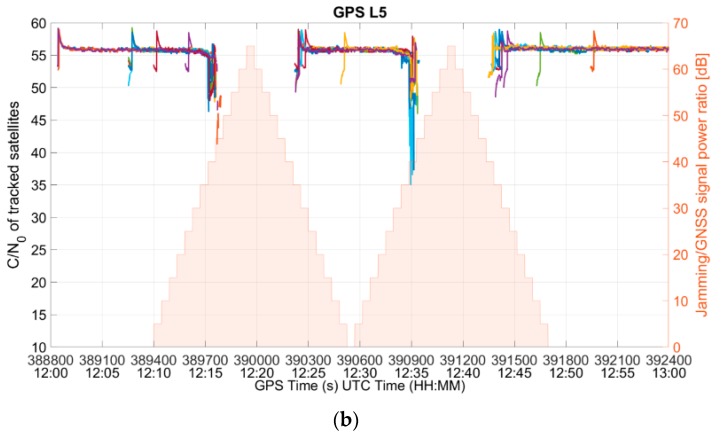
C/N_0_ of (**a**) GPS L1, and (**b**) GPS L5 tracked satellites.

**Figure 12:**
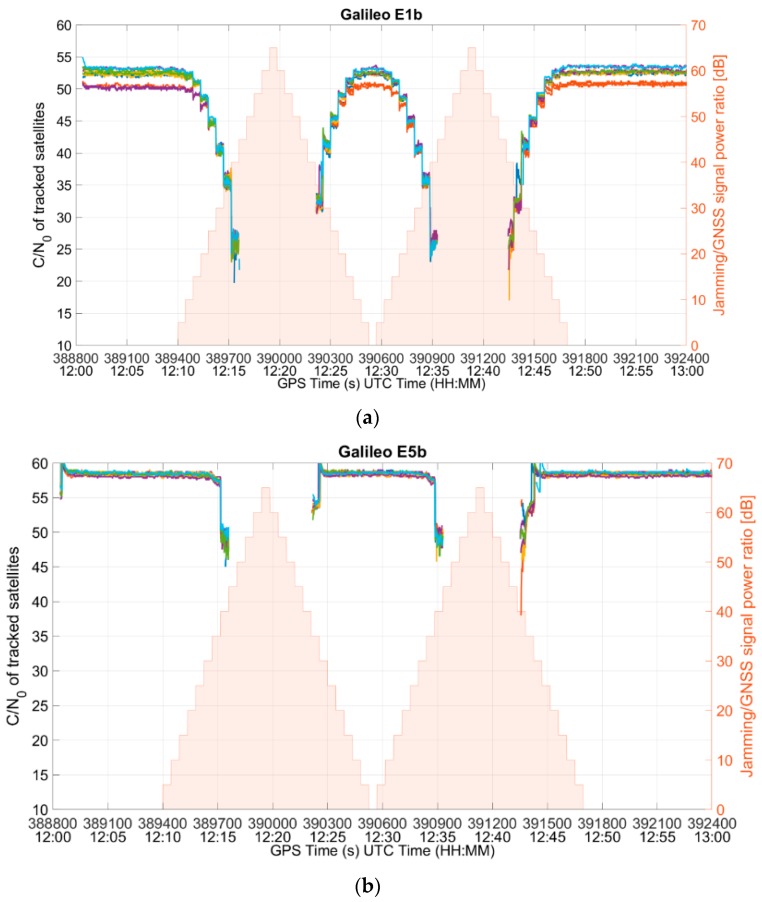
C/N_0_ of (**a**) Galileo E1b, and (**b**) Galileo E5b tracked satellites.

Since no information on the L5/E5 signals could be retrieved from the NMEA files, the analysis was conducted using a MATLAB-based script that processes the RINEX files from the RUT. For each of the five signals (GPS L1, GPS L5, Galileo E1, Galileo E5b, and Galileo E5a), the number of tracked satellites and the signal strength from the tracked satellites for each signal were analyzed. The impact of the interference on L1/E1 band is clearly visible in Figure 11a and Figure 12a, which show the C/N_0_ values for the GPS L1 C/A and the Galileo E1b signals, respectively. As expected, as the interference power increases, the C/N_0_ ratio of the signals in the L1/E1 band decreases until, at some point, the RUT stops tracking them and it is no longer offering the corresponding observations. It is interesting to find out that the signals in the L5/E5 band are also affected, even though the frequencies are far apart from the interfering band (i.e., Figure 11b and Figure 12b).

However, it can also be observed that the receiver does not generate L5/E5 observations for those epochs in which no L1/E1 observation is generated. It can be seen from both Figure 11 and Figure 12 that the data gaps in the test case for both the frequencies were identical. No observation is generated for GPS L5 satellites as soon as the L1 signals are so degraded that the RUT cannot track them, even though the L5 signal quality is still good. The same happens with Galileo. This shows that the RUT was not yet able to exploit the frequency diversity to withstand the interference. 

## 7. Conclusions

The results of the standardized tests for the two categories of receivers were presented. The results were analyzed in terms of well-defined receiver key performance indicators. The result analysis showed that both the receiver categories were impacted ominously by the interference threats. More specifically, it was observed that the mass-market RUT was capable of recovering from a high-powered jamming event much faster than the professional grade RUT. Moreover, the MM RUT was capable of withstanding a power-varying interference event longer than the PRO RUT, resulting in a much higher availability for MM RUT than that of a PRO RUT. On the contrary, in terms of performance accuracy, PRO RUT is instead performing better than the MM RUT. However, when a C/N_0_ mask of 25 dB-Hz was applied at the PVT computation stage for both the receiver categories, they tend to behave almost similarly against interference threats, i.e., both the receivers exhibit similar availability against the same kind of interference threat. 

A performance comparison of the RUT under different threat signatures, considering the methodology the interfering signal is generated (i.e., via synthetic signal or via real recorded signal), was also presented. The objective of this test set up was to investigate the impact of the interference signal generation on the RUT’s positioning performance. The results showed that the interfering signals generated synthetically had impacted the RUT more than the real-recorded version of the same signatures. This could potentially be due to the fact that the synthetic signatures are free from multipath and GNSS PRN codes and, hence, are much cleaner than the recorded ones, resulting in a far-reaching impact on the RUT’s navigation performance. 

Dual frequency GPS/Galileo L1/E1 and L5/E5 test was also carried out in order to investigate if the RUT could intelligently exploit the frequency diversity in the presence of interference in the L1/E1 frequency band. It was interesting to experience that the L5/E5 signals were also affected to a lesser extent, even though the interference was on L1/E1 band. It was noticed that the professional RUT did not generate L5/E5 observations for those epochs in which no L1/E1 observation was generated. Based on this result, it can be stated that the RUT was not yet capable of exploiting the frequency diversity to withstand the interference in L1/E1 band.

Overall, this paper shows that the availability of threat signatures and standardized test procedures is critical towards understanding the behavior of GNSS receivers under the most frequently encountered interference threats. This, in turn, should help the GNSS community in developing the next generation of robust receiver technologies and to support wider utilization of GNSS in safety and liability-critical high-value applications.

## Figures and Tables

**Table 1:** Simulated scenario settings.

**Constellation**	GPS + Galileo
**Number of satellites**	10 (GPS) + 10 (Galileo)
**Centre frequency (MHz)**	1575.42
**Ionosphere model**	Klobuchar (default parameters)
**Troposphere model**	Saastamoinen (default parameters)
**Start time**	01.02.2018-12:00:00
**Duration (min)**	30 (TTRP) / 60 (sensitivity test and baseline)
**GNSS signal power (dBm)**	−125
**Interference power level for** **‘TTRP test method’ (dBm)**	−35
**Interference power range for** **‘Sensitivity test method’ (dBm)**	[−120: 5: −60]
**J/S range for ‘Sensitivity test method’ (dB)**	[5: 5: 65]
**Receiver location (Lat/Long/Alt)**	60°N/24°E/30 m

**Table 2:** Receiver configuration.

**C/N_0_ threshold for satellite in Navigation Computation (dB-Hz)**	25
**Minimum elevation angle**	5°
**Start mode**	Cold start
**Dynamic model**	Stationary

**Table 3:** Description of baseline set of selected threats.

Threat Id	Type of Signal	Example Plot	Reason for Choice
01	Wide Sweep-fast repeat rate	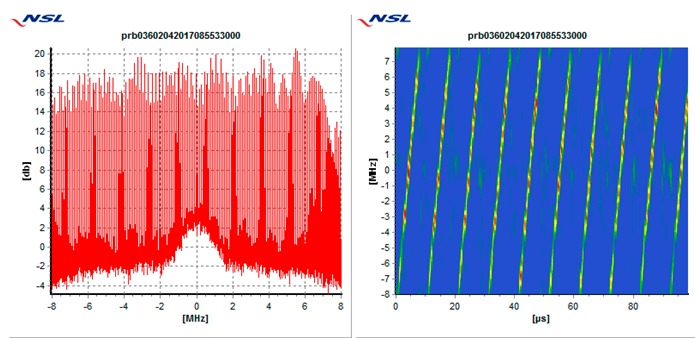	Very common (total number of events, and number of sites)
02	Multiple narrowband	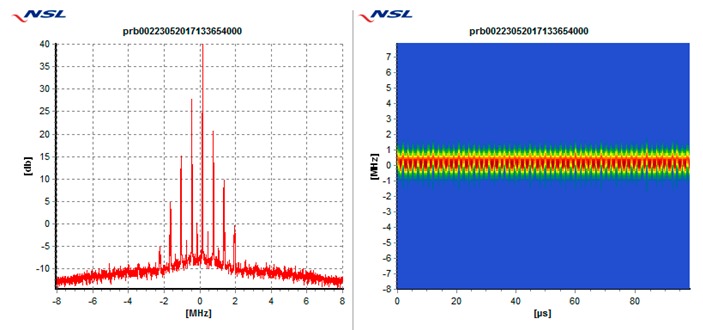	Very common (total number of events and number of sites)
03	Triangular	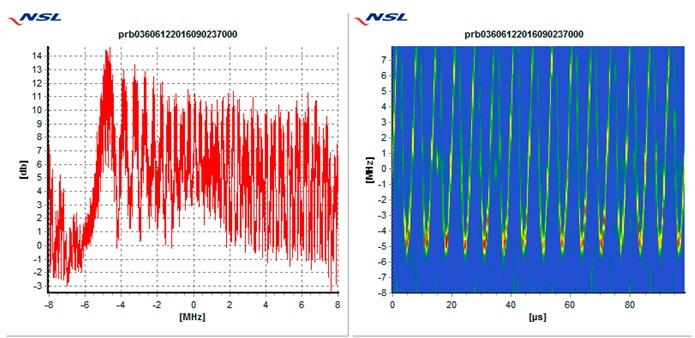	Common (number of sites)
04	Triangular wave	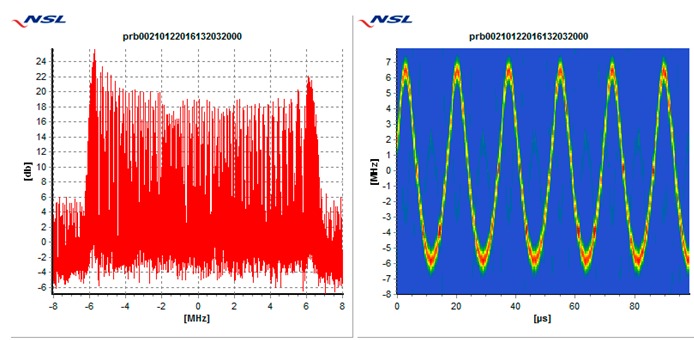	Common (number of sites)
05	Tick	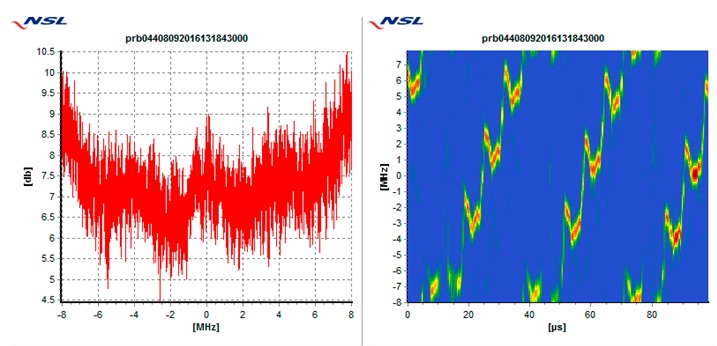	Quite common. Evolving threat (new type)

**Table 4:** Test results for MM RUT.

Test Case	Maximum Horizontal Position Error (m)	Maximum Vertical Position Error (m)	Position Fix Availability	J/S_PVT_lost_ (dB)	J/S_PVT_reobtained_ (dB)	TTRP * (s)
Baseline	0.74	1.7	100%	-	-	-
MM01	6.14	8.3	65.07%	50	35	4
MM02	5.11	4.5	67.96%	50	45	1
MM03	40.30	32.28	60.44%	45	35	1
MM04	5.68	3.7	61.47%	50	30	1
MM05	3.9	3.1	70.27%	50	45	1

* TTRP values are otained with TTRP test cases and the other matrices (e.g., maximum horizontal position error, maximum vertical position error, etc.) are obtained either in the baseline test case or in different sensitivity test cases, as represented by the test id.

**Table 5:** Test results for PRO RUT.

Test Case	Maximum Horizontal Position Error (m)	Maximum Vertical Position Error (m)	Position Fix Availability	J/S_PVT_lost_ (dB)	J/S_PVT_reobtained_ (dB)	TTRP * (s)
Baseline	0.28	0.41	100%	-	-	-
PRO01	3.76	1.89	66.36%	55	35	9
PRO02	0.66	1.88	66.09%	55	35	10
PRO03	0.97	2.19	66.04%	45	35	10
PRO04	0.78	0.97	63.16%	40	45	6
PRO05	2.63	1.55	61.29%	40	40	6

* TTRP values are otained with TTRP test cases and the other matrices (e.g., maximum horizontal position error, maximum vertical position error, etc.) are obtained either in baseline test case or in different sensitivity test cases, as represented by the test id.

**Table 6:** Test results for MM and PRO RUT when default C/N_0_ settings are used.

	Maximum Horizontal Position Error (m)		Maximum Vertical Position Error (m)		Position Fix Availability		J/S_PVT_lost_ (dB)
	Test Case	Baseline	Test Case	Baseline	Test Case	Baseline	
Mass market RUT	78.8	0.74	167.4	1.7	97.91%	100%	30 (on the falling ramp)
Professional grade RUT	0.72	0.28	0.78	0.41	58.58%	100%	45 (on the rising ramp)

**Table 7:** Test results for MM RUT when with synthetic and recorded interference signals are used.

	Maximum Horizontal Position Error (m)		Maximum Vertical Position Error (m)		Position Fix Availability		J/S_PVT_lost_ (dB)		J/S_PVT_reobtained_ (dB)	
	S	R	S	R	S	R	S	R	S	R
MM01	0.44	6.14	1.3	8.3	43.16%	65.07%	45	50	10	35
MM02	3.12	5.11	3.2	4.5	50.98%	67.96%	50	50	25	45
MM03	0.81	40.3	2	32.28	41.47%	60.44%	50	45	10	35
MM04	1	5.68	2.1	3.7	39.91%	61.47%	55	50	0	30
